# Borderline personality disorder symptoms moderate the effect of risk-legitimizing beliefs on risky motorcycle riding among Thai university students

**DOI:** 10.3389/fpubh.2026.1759240

**Published:** 2026-02-19

**Authors:** Xu Quan, Chawisa Suradom, Tinakon Wongpakaran, Justin DeMaranville

**Affiliations:** 1 Mental Health Program, Multidisciplinary and Interdisciplinary School (MIdS), Chiang Mai University, Chiang Mai, Thailand; 2 Department of Psychiatry, Faculty of Medicine, Chiang Mai University, Chiang Mai, Thailand

**Keywords:** borderline personality disorder symptoms, public mental health, risk-legitimizing beliefs, risky motorcycle riding, university students

## Abstract

**Background:**

Motorcycle crashes remain a significant public health concern in Thailand, particularly among young adults who frequently engage in risky riding. Cognitive justifications—termed risk-legitimizing beliefs (RLBs)—may normalize unsafe behaviors, whereas borderline personality disorder (BPD) symptoms could intensify such tendencies. This study examined whether BPD symptoms moderate the association between RLBs and risky motorcycle riding.

**Materials and methods:**

A cross-sectional online survey was conducted among 247 university student motorcyclists in Northern Thailand. Participants completed validated Thai versions of the Motorcycle Rider Behavior Questionnaire (MRBQ), a 13-item RLBs scale, and the Screening Instrument for Borderline Personality Disorder (SI-Bord).

**Results:**

Approximately 23.5% of the participants scored above the SI-Bord cut-off (>7), indicating that clinically relevant BPD symptoms were relatively common in this sample. Hierarchical regression analyses tested whether BPD symptoms moderate the association between RLBs and risky riding, adjusting for demographic and behavioral covariates. Both RLBs (*β* = 0.246, *p* < 0.001) and BPD symptoms (*β* = 0.293, *p* < 0.001) independently predicted higher risky riding, and their interaction was significant (*β* = 0.212, *p* < 0.001). The complete model explained 38.1% of the variance.

**Conclusion:**

Cognitive rationalizations and personality vulnerability jointly contribute to unsafe motorcycle riding. Preventive efforts should identify riders with elevated BPD symptoms and combine belief modification, emotion-regulation training, and peer-norm interventions to reduce motorcycle-related risks among young adults, especially in low- and middle-income settings.

## 1 Introduction

Road traffic crashes represent a persistent global public health and medical crisis. According to the Global Status Report on Road Safety (1), an estimated 1.19 million people died from road traffic injuries in 2021, with nearly 90% of fatalities occurring in low- and middle-income countries (LMICs). Road traffic injuries remain the leading cause of death among individuals aged 5–29 years. Motorcyclists are disproportionately vulnerable, accounting for 48% of road traffic deaths in Southeast Asia compared with 21% globally (1). In Thailand, motorcycles are involved in approximately 90% of traffic-related injuries, and riders aged ≤24 years consistently represent more than one-third of all cases (2). The Global Burden of Disease 2021 study further identified road traffic injuries as among the top contributors to disability-adjusted life years (DALYs) in Thailand, underscoring the high mortality and long-term disability burden (3). These figures highlight the societal toll of motorcycle-related injuries and illuminate the urgent need to understand the psychological and behavioral mechanisms underlying unsafe riding.

A key psychological explanation for unsafe riding or driving has traditionally focused on risk perception, defined as individuals’ subjective assessments of the likelihood and severity of potential harm (4, 5). Risk perception has been widely examined in traffic safety research (6, 7); however, empirical findings regarding its association with risky riding or driving behaviors have been mixed. While some studies report that higher perceived risk is associated with safer behavior (6, 8, 9), others find weak or non-significant relationships (10). Importantly, a recurring phenomenon in traffic safety is that individuals may be aware of the dangers of riding behaviors—such as speeding or risky maneuvering—yet continue to engage in them (11). This discrepancy between risk awareness and persistent risky behavior suggests that risk perception alone may be insufficient to explain why unsafe riding is maintained, highlighting the need to consider additional psychological mechanisms beyond risk appraisal.

To address this gap, the study focused on risk-legitimizing beliefs (RLBs). These beliefs provide cognitive rationalizations that make risky practices appear acceptable. Prior work on related health-risk behaviors (e.g., smoking) suggests that such rationalizations commonly involve beliefs that emphasize short-term functional benefits (e.g., saving time or reducing stress) and beliefs that minimize perceived harm or overestimate control. When individuals recognize the dangers of their actions but continue to engage in them, they experience psychological discomfort known as cognitive dissonance (12). RLBs may serve to reduce such cognitive dissonance by reframing risky behavior as acceptable or justified. Although RLBs are related to constructs such as optimism bias and the illusion of control, they differ in their primary functional focus in regulating risky behavior. Optimism bias refers to unrealistically favorable judgments about one’s personal risk relative to others (7), and illusion of control reflects overestimation of one’s ability to control outcomes (13, 14); both primarily influence how risk or control is construed or perceived. By contrast, RLBs are defined here as belief tendencies that justify and sustain engagement in risky riding, particularly in situations where potential risk is acknowledged, but behavior is maintained. In this sense, RLBs emphasize the perceived acceptability of continued risky behavior, rather than the appraisal of risk itself. Consistent with this conceptualization, evidence from smoking research has shown that RLBs are associated with continued engagement in harmful behaviors and reduced motivation to change (15, 16). Yet systematic investigation of RLBs in motorcycle riding remains limited, particularly among university students in LMICs.

From a public mental health perspective, adolescence and young adulthood represent developmental stages marked by heightened vulnerability to mental health problems and risk-taking behaviors (17–19). Borderline personality disorder (BPD) symptoms typically emerge in early adulthood—a period encompassing many university students. Characterized by affective instability, impulsivity, and interpersonal difficulties (20), these symptoms may impair judgment, lower risk perception, and heighten susceptibility to dangerous behaviors such as risky motorcycle riding (21, 22).

Both the Diagnostic and Statistical Manual of Mental Disorders, Fifth Edition, Alternative Model for Personality Disorders (DSM-5 AMPD) (20) and the International Classification of Diseases, 11th Revision (ICD-11) (23) emphasize that personality pathology exists along a continuum rather than as a categorical disorder. In DSM-5, impulsivity across potentially self-damaging domains—including reckless driving—is recognized as a core feature of BPD. Pooled estimates indicate that approximately 10% of university students meet criteria for clinically significant BPD (24), whereas subthreshold BPD features are also frequently observed in university populations (25) and may confer behavioral risk even below diagnostic thresholds.

Despite this relevance, empirical evidence linking BPD symptoms to driving and riding outcomes remains limited. Existing studies suggest that individuals with BPD traits are more prone to traffic violations and aggressive driving (26, 27), yet most research has focused on clinical or offender samples, limiting generalizability to community riders. Data from nonclinical university populations, especially in Southeast Asia, are scarce. Given that motorcycles account for a disproportionate share of road injuries in Thailand, examining how BPD symptoms relate to risky riding behaviors among university motorcyclists represents an important yet underexplored area of research.

The intersection between cognitive beliefs and personality vulnerabilities offers a critical but understudied perspective on risk behavior. The presence of BPD symptoms may amplify the influence of RLBs on hazardous motorcycle riding, with implications for both mental and physical health. However, few studies have examined how public mental health factors, particularly BPD symptoms, interact with cognitive beliefs about risk to influence dangerous riding among Thai university students—an LMIC context with a disproportionately high burden of motorcycle injuries. Addressing this gap is essential for developing integrated interventions that consider both psychological predispositions and behavioral risk factors.

Accordingly, the present study investigated whether BPD symptoms moderate the association between RLBs and risky motorcycle riding in a nonclinical cohort of university motorcyclists in Northern Thailand. Based on prior theory and empirical evidence, the following hypotheses were tested:

*H*1: Higher levels of RLBs are associated with greater risky motorcycle riding behaviors.

*H*2: Higher levels of BPD symptoms are associated with greater risky motorcycle riding behaviors.

*H*3: BPD symptoms moderate the association between RLBs and risky motorcycle riding, such that the association is stronger at higher levels of BPD symptoms.

**Figure 1:**
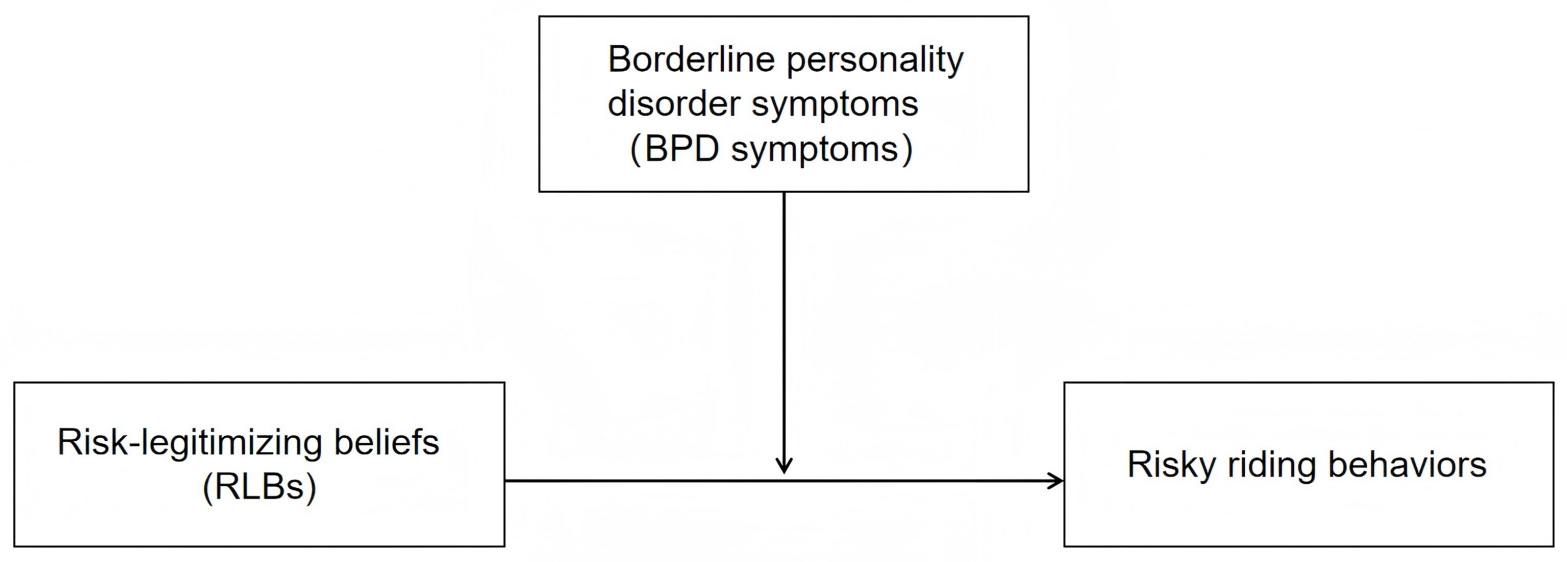
Research framework of the study.

The overall research framework and hypothesized relationships are illustrated in Figure 1. Additionally, previous research has identified several demographic and behavioral risk factors—such as younger age, being male, limited riding experience, frequent motorcycle use, alcohol consumption, and lack of a valid driving license—that have been consistently linked to higher crash risk among young riders (28–32). Recognizing these established influences, the present study sought to statistically control for them to better isolate the psychological mechanisms of interest. By focusing on the intersection of risk behaviors and public mental health, this study aims to inform more comprehensive prevention strategies that address both unsafe riding and the underlying psychological vulnerabilities contributing to it.

## 2 Materials and methods

### 2.1 Study population and sample

This study employed a cross-sectional online survey among university motorcycle riders in Northern Thailand (Chiang Mai province). Participants were recruited via convenience sampling from verified Facebook student groups that required student ID verification for membership. Eligible participants were full-time university students aged 20–24 years who used motorcycles as their primary mode of transport in Chiang Mai. Those engaged in professional riding activities (e.g., ride-hailing or delivery services) were excluded. A total of 318 students accessed the survey.

### 2.2 Procedures

The study protocol was approved by the Research Ethics Committee, Faculty of Medicine, Chiang Mai University (protocol code: PSY-2567-0577). Best-practice guidance for reporting web surveys (e.g., the Checklist for Reporting Results of Internet E-Surveys [CHERRIES]) (33) was followed to ensure methodological transparency and data quality. The Thai-language questionnaire was hosted on Microsoft Forms and optimized for mobile devices. The anonymous online format, voluntary participation, and assurance of confidentiality were expected to minimize social desirability bias when reporting sensitive behaviors such as risky riding. A pretest was conducted with 38 university students (not included in the final sample) to assess item clarity, cultural relevance, and technical functionality.

Recruitment posts, including a standardized invitation and flyer with a survey link and QR code, were shared in Facebook groups of universities and student communities in Chiang Mai during November–December 2024. The survey landing page displayed the participant information sheet and electronic informed consent; only those who provided consent could proceed. Upon verified completion, participants received 50 THB (≈1.50 USD). Compensation records were kept separate from survey data to preserve confidentiality. All data were securely stored on password-protected university servers, with access restricted to the study team.

Because recruitment occurred in university social-media groups, it was not possible to determine how many individuals viewed the invitation posts—a typical limitation of online recruitment. Details of data-quality screening procedures are provided in Section 2.4.

### 2.3 Measurements

#### 2.3.1 Risky riding behaviors

Risky riding behaviors were assessed using a modified Thai version of the Motorcycle Rider Behavior Questionnaire (MRBQ) (34–36). Participants were instructed to indicate how often they had engaged in each behavior during the past 12 months. The questionnaire captured common riding errors (e.g., misjudging gaps, distraction) and violations (e.g., speeding, aggressive overtaking). Responses were rated on a six-point Likert scale (1 = Never to 6 = All the time), with higher scores indicating greater involvement in risky riding.

Although the MRBQ comprises multiple behavioral domains, some prior applications have treated the items collectively when examining overall riding or risky-riding tendencies [e.g., (37–39)], and the present study followed this analytic approach. To derive a composite indicator of overall risky-riding propensity, a principal component analysis (PCA) with fixed one-factor extraction was performed. Sampling adequacy was acceptable (KMO = 0.834; Bartlett’s test χ^2^ = 2156.554, *p* < 0.001). Twenty-six items were retained, each demonstrating a positive loading on the general factor (range = 0.419–0.619). Consistent with this pattern, the scree plot showed a marked drop after the first eigenvalue, supporting the use of a single total score indexing overall risky-riding propensity in this sample. The resulting composite demonstrated excellent internal consistency (Cronbach’s *α* = 0.881).

#### 2.3.2 Risk-legitimizing beliefs

Risk-legitimizing beliefs (RLBs) were measured using a 13-item scale developed for this study, adapted from previously validated instruments on risk rationalization and driving beliefs (9, 40). The items were reviewed independently by three subject-matter experts for conceptual clarity and relevance to risk rationalization, pilot-tested with 38 university students for face validity, and refined for cultural appropriateness. Expert feedback confirmed that all items were clearly understood and aligned with the intended construct. Participants rated each item on a five-point Likert scale (1 = Strongly disagree to 5 = Strongly agree), with higher scores indicating stronger endorsement of RLBs.

Given the exploratory nature of the study and the preliminary status of the adapted items, PCA was conducted to assess whether all items reflected a single underlying construct. The scree plot showed a dominant first factor, and communalities for most items exceeded 0.40, indicating substantial shared variance. Although one item demonstrated a lower communality, the overall pattern supported essential unidimensionality. Treating RLBs as a single composite was therefore considered appropriate for the aims of the present study, which focused on examining belief-based rationalization as a general psychological tendency rather than on differentiating specific belief subtypes. The final 13-item composite demonstrated good internal consistency (Cronbach’s *α* = 0.821).

#### 2.3.3 Screening instrument for borderline personality disorder

Borderline personality disorder (BPD) symptoms were assessed using the Screening Instrument for Borderline Personality Disorder (SI-Bord) (41). Participants rated their experiences on a four-point Likert scale (0 = Never to 3 = Very often), with total scores ranging from 0 to 15. Higher scores indicated greater severity of BPD symptoms. A cut-off score > 7 indicates clinically significant BPD symptoms. In this study, the SI-Bord demonstrated satisfactory internal consistency (Cronbach’s *α* = 0.719).

#### 2.3.4 Perceived peers’ risky riding

Perceived peers’ risky riding was assessed using items from Lim et al. (10). Participants rated how frequently they perceived their peers engaging in risky riding behaviors on a five-point Likert scale (0 = Never to 4 = Very often). Higher scores indicated stronger perceptions of peer involvement in risky riding. Internal consistency was excellent (Cronbach’s *α* = 0.834).

#### 2.3.5 Covariates

Demographic and riding-related variables were included as covariates in the analysis. These comprised gender (male/female), age (in years), riding experience (in years), riding frequency (days per week), possession of a valid driving license (yes/no), history of alcohol use (yes/no), and history of mental conditions (yes/no). All covariates were self-reported and have been shown in prior studies to influence risky riding behaviors among riders.

Different response formats were intentionally used for each construct to reflect their theoretical distinctions and established measurement traditions: symptom-based ratings for BPD features, frequency ratings for risky riding behaviors, and Likert-type agreement ratings for RLBs. All instruments were drawn from, or adapted based on, previously validated measures. For each scale, we used the total score (calculated as the sum of all item responses) to represent the relevant construct, as each scale is unidimensional and measures a single underlying factor. Our analyses focused on examining associations between constructs rather than making direct comparisons of raw scores across different measures. To address potential issues related to scale heterogeneity, we report and interpret standardized coefficients (beta weights) from hierarchical regression analyses. This approach facilitates meaningful comparison of the relative importance of each construct, regardless of their original scale formats, and minimizes the risk of method bias.

### 2.4 Data screening and preparation

Of the 318 visitors who accessed the online survey, 284 provided electronic consent and completed the questionnaire. Data-quality screening followed prespecified criteria to enhance transparency and reproducibility: (a) completion time—responses submitted in less than approximately one third of the pilot median duration (median ≈17 min; < 6 min; 16 cases). This cut-off was derived from the pretest completion-time distribution, consistent with CHERRIES (33) requirements to justify exclusion criteria for atypically fast submissions; (b) response patterns indicating straight-lining or logical inconsistencies across demographic and behavioral items (14 cases); and (c) duplicate entries identified through submission timestamps, anonymized IP patterns, and compensation records, with no personally identifiable data retained (4 cases).

All variables were subsequently inspected for missing data, normality, and outliers prior to analysis. Three cases exceeding ±3.0 SD on the risky riding composite were excluded as univariate outliers. Missing values within multi-item scales were handled by computing the mean of available items when at least 80% of items were completed, following established psychometric guidelines. Missingness was minimal (<2%) at the scale level; therefore, no additional imputation was performed. After all exclusions, the final analytic sample comprised 247 participants.

Sample size adequacy was evaluated with reference to established guidelines for multiple regression analyses. Green (42) proposed practical rules-of-thumb suggesting a minimum sample size of N ≥ 50 + 8 m for testing overall model fit and N ≥ 104 + m for testing individual predictors, where m represents the number of predictors included in the model. In the present study, 11 predictors were entered into the regression models (including the interaction term), yielding minimum recommended sample sizes of *N* = 138 and *N* = 115, respectively. The final sample size (*N* = 247) exceeded both thresholds, supporting the adequacy of the sample for the regression analyses conducted.

### 2.5 Statistical analysis

All analyses were conducted using IBM SPSS Statistics (Version 27). Descriptive statistics were calculated for all study variables. For multi-item scales, mean (M), standard deviation (SD), and internal consistency (Cronbach’s *α*) were reported. For categorical variables, frequency and percentage (n/%) were presented. Bivariate associations among continuous variables were examined using Pearson’s r. For all constructs measured using multiple items, total scores were calculated by summing item responses, with higher scores indicating higher levels of the corresponding construct. Prior to correlation analyses, internal consistency was assessed for each scale, and all composite measures demonstrated acceptable reliability. The psychometric properties of the adapted RLBs scale were further examined using exploratory factor analysis, as described above. Correlation analyses were then conducted using these summed total scores.

Hierarchical linear regression was employed to test whether BPD symptoms moderated the association between RLBs and risky riding. Standardized coefficients (*β*), t-values, and change statistics (ΔR^2^, ΔF) were reported. Simple-slopes analyses and the interaction plot were generated using the PROCESS macro for SPSS (Model 1) based on the same regression outputs. Statistical significance was set at *p* < 0.05 (two-tailed). As part of regression diagnostics, key model assumptions were examined. Visual inspection of residual plots suggested no major deviations from linearity or homoscedasticity, and residuals appeared approximately normally distributed. No influential outliers were identified based on standardized residuals and leverage values.

## 3 Results

### 3.1 Descriptive statistics of sample characteristics

As shown in Table 1, the analytic sample comprised 247 university motorcycle riders (M = 21.15 years, SD = 1.21), the majority of whom were female (72.9%) and enrolled in bachelor’s degree programs (96.0%). Riding exposure was substantial, with over half (52.2%) reporting daily riding and another 28.7% riding almost every day. Most participants had at least 1 year of riding experience; 37.7% reported 5–10 years. Motorcycles with engine capacities of 125 cc or lower were most common (72.5%). A large majority (81.4%) held a valid driving license, and 70.4% reported alcohol use. Regarding mental health, 9.7% of participants reported a history of diagnosed mental disorders.

**Table 1:** Descriptive statistics of sample characteristics.

Characteristics (*n* = 247)		*n* (*%*) or *M* (*SD*)
Gender	Female	180 (72.9)
	Male	67 (27.1)
Age (years)		21.15 ± 1.21
Education	Bachelor’s degree	237 (96.0)
	Master’s degree	10 (4.0)
Riding frequency	Sometimes	22 (8.9)
	Often	25 (10.1)
	Almost every day	71 (28.7)
	Always	129 (52.2)
Riding experience	0 to 1 year	21 (8.5)
	1 to 3 years	49 (19.8)
	3 to 5 years	62 (25.1)
	5 to 10 years	93 (37.7)
	More than 10 years	22 (8.9)
Size of motorcycle	125 cc or lower	179 (72.5)
	More than 125 cc	68 (27.5)
Status of driving license	No	46 (18.6)
	Yes	201 (81.4)
Status of drinking	No	73 (29.6)
	Yes	174 (70.4)
History of mental disorders	No	223 (90.3)
	Yes	24 (9.7)

Table 2 summarizes the descriptive statistics of the key study variables. Participants reported moderate levels of risky riding (M = 42.55, SD = 11.59), with positive skewness suggesting that a subgroup of riders engaged in relatively high levels of risky behavior. Risk-legitimizing beliefs (RLBs) were common (M = 33.34, SD = 8.26), and BPD symptoms were present at sub-clinical but meaningful levels (M = 5.14, SD = 3.14). Perceived peers’ risky riding was generally low (M = 9.87, SD = 5.90) but highly skewed, reflecting that a minority perceived their peers as highly risky. Approximately 23.5% of participants scored above the SI-Bord cut-off (>7), indicating clinically relevant levels of BPD symptoms.

**Table 2:** Descriptive statistics of the measurements.

Measurement (score range)	Mean ± SD	Skewness	Kurtosis
Risky riding behaviors (26–156)	42.55 ± 11.593	1.020	1.265
Risk-legitimizing beliefs (13–65)	33.34 ± 8.259	0.027	−0.105
BPD symptoms (0–15)	5.14 ± 3.139	0.410	−0.211
Perceived peers’ risky riding(0–52)	9.87 ± 5.904	1.397	2.763

Table 3 presents the mean endorsement of each RLBs item, ranked in descending order of mean value. The most strongly endorsed beliefs were “Even when I’m very tired, I ride my motorcycle to avoid being late for classes or important events” (M = 3.78, SD = 1.16) and “Riding fast allows me to reach my destination more quickly” (M = 3.45, SD = 1.21), reflecting functional rationalizations that emphasize short-term benefits. Other items showed comparatively lower endorsement levels. These patterns suggest that interventions should prioritize addressing time-saving and control-related justifications, which appear most salient among university riders.

**Table 3:** Mean and standard deviation of risk-legitimizing beliefs (RLBs) items (*N* = 247).

Item	M	SD
1. Even when I’m very tired, I ride my motorcycle to avoid being late for classes or important events.	3.78	1.160
2. Riding fast allows me to reach my destination more quickly.	3.45	1.205
3. For short trips, wearing a helmet does not seem necessary.	2.94	1.150
4. I sometimes avoid wearing a helmet because it feels uncomfortable.	2.89	1.311
5. Even when I’m very tired, I believe I can still ride safely based on my experience.	2.87	1.199
6. I am able to stay focused while riding, even with distractions.	2.77	1.136
7. Riding a motorcycle fast gives me a thrill.	2.47	1.343
8. I feel confident riding faster on familiar roads.	2.47	1.096
9. It’s not dangerous to ride a motorcycle fast when there are no other vehicles around.	2.26	1.078
10. After drinking alcohol, I still feel capable of riding a motorcycle.	2.17	1.153
11. I feel confident riding at higher speeds because of my experience.	2.03	1.098
12. I feel the need to stay connected with work, friends, or family through my phone, even while riding my motorcycle.	1.68	0.961
13. Riding after drinking alcohol makes me feel more confident and energized.	1.55	0.867

Items are listed in descending order of mean value, with higher scores indicating stronger agreement.

A summary of item-level risky riding behaviors is shown in Table 4. Phone- or earphone-related distractions (Item 1) and fatigue-related behaviors such as riding long periods without breaks (Item 2) had the highest mean frequencies, indicating that everyday inattention and physical strain were the most common sources of risky riding in this sample. Moderate frequencies were observed for routine traffic violations—including late-night speeding, failing to stop at zebra crossings, and riding too close to other vehicles (Items 3–5)—suggesting that rule-breaking behaviors were present but less frequent than distraction- or fatigue-related behaviors. Deliberate high-risk actions such as wheel spins or lifting the front wheel (Items 25–26) were reported least often, indicating that intentional stunt-like behaviors were relatively rare among university riders. These patterns suggest that the risky riding observed in this sample is driven primarily by common, everyday behaviors rather than extreme forms of risk-taking.

**Table 4:** Mean and standard deviation of items in risky riding behaviors (*N* = 247).

Item	M	SD
1. You engage in distractions involving your phone or earphones while riding.	2.65	1.595
2. You ride for long periods without taking breaks.	2.36	1.207
3. Breaking speed limits at late night or early morning.	2.21	1.303
4. Not stopping the motorcycle at zebra crossings to let pedestrians cross the road.	2.12	0.972
5. Riding too close to the car in front. If there is an emergency, it will be difficult to stop.	2.08	1.044
6. Overtaking in an overtaking-prohibited area.	2.04	1.049
7. Overtaking without giving a signal in advance.	2.00	1.067
8. Failing to control the motorcycle when using high speed.	1.98	1.124
9. You start riding even though you feel very sleepy.	1.94	1.078
10. Parking your motorcycle in a prohibited area.	1.93	1.004
11. Riding without caution about any vehicle pulling out in front of you and having difficulty stopping.	1.91	1.000
12. Turning into a side street from a main road without being wary of any pedestrians.	1.89	0.874
13. Failing to notice a car from behind a parked vehicle until it is nearly too late.	1.88	0.869
14. Riding too fast into a corner or junction.	1.80	0.986
15. You read or type messages on your phone while riding.	1.75	0.963
16. Ignoring the “GIVE WAY” sign when driving on narrow roads and not letting a driver from the other lane proceed.	1.67	0.925
17. Violating red traffic lights.	1.63	0.825
18. You ride after drinking alcohol, especially during major festivals.	1.55	1.018
19. Driving against the lane direction or the wrong way on a one-way street.	1.53	0.805
20. Turning left into a main road without reducing speed and not noticing other vehicles.	1.50	0.686
21. Getting involved in unofficial “races” starting from traffic light junctions.	1.47	0.999
22. You check or scroll through social media while riding.	1.47	0.830
23. If there are no cars on the road, you continue to go straight while ignoring red traffic lights.	1.42	0.812
24. Cutting other vehicles off/overtaking at close range.	1.30	0.648
25. Purposely doing a wheel spin.	1.10	0.440
26. Pulling away too quickly and your front wheel lifts off the road.	1.03	0.210

### 3.2 Correlation analysis

Correlations among all study variables are presented in Table 5. Risky riding behaviors were positively correlated with RLBs (*r* = 0.395, *p* < 0.01), BPD symptoms (*r* = 0.330, *p* < 0.01), and perceived peers’ risky riding (*r* = 0.416, *p* < 0.01). These associations indicate that students who more strongly endorsed RLBs or reported higher BPD symptoms tended to engage in more risky riding behaviors. In addition, RLBs were significantly correlated with perceived peers’ risky riding (*r* = 0.319, *p* < 0.01) but were not significantly related to BPD symptoms (*r* = 0.061, *p* > 0.05). All correlations were in the expected directions and of moderate magnitude, suggesting minimal multicollinearity among predictors for subsequent regression analyses.

**Table 5:** Correlation between variables and risky riding behaviors.

Variables	1	2	3	4	5	6	7	8	9	10	11	12
1. Gender	--											
2. Age	0.058	--										
3. Riding frequency	0.197**	−0.029	--									
4. Riding experience	0.184**	0.257**	0.424**	--								
5. Size of motorcycle	0.069	0.013	−0.106	0.004	--							
6. Status of riding license	0.105	−0.112	0.056	0.127*	−0.032	--						
7. Status of drinking	0.036	−0.035	0.182**	0.077	−0.020	−0.059	--					
8. History of mental disorders	−0.169**	0.072	0.017	0.019	−0.062	−0.019	−0.117	--				
9. RLBs^1^	0.166**	−0.095	0.142*	0.099	−0.041	0.129*	0.193**	−0.053	--			
10. BPD^2^ symptoms	−0.033	0.020	−0.055	−0.031	0.014	−0.005	0.001	0.286**	0.061	--		
11. Perceived peers’ risky riding	0.164**	−0.027	0.213**	0.100	0.026	0.070	0.094	−0.046	0.319**	0.088	--	
12. Risky riding behaviors	0.166**	0.042	0.246**	0.103	−0.034	0.076	0.130*	0.023	0.395**	0.330**	0.416**	--

**p < 0.01, *p < 0.05.

1 Risk-legitimizing beliefs.

2 Borderline personality disorder.

### 3.3 Moderation analysis

The moderation model was tested using mean-centered scores for RLBs (X) and BPD symptoms (W), with their interaction term (X × W) entered in the second step of hierarchical regression. The detailed standardized coefficients and confidence intervals for each model are presented in Table 6, and the overall model fit indices are summarized in Table 7.

**Table 6:** The moderating effect of BPD symptoms between RLBs and risky riding behaviors.

Model		*β*	t	*p-*value	LLCI	ULCI
1	Constant		66.342	<0.001	41.305	43.833
R^2^ = 0.250	RLBs (X)	0.376	6.767	<0.001	0.374	0.681
	BPD symptoms (W)	0.307	5.521	<0.001	0.729	1.537
2	Constant		67.525	<0.001	41.203	43.679
R^2^ = 0.285	RLBs (X)	0.373	6.866	<0.001	0.373	0.674
	BPD symptoms (W)	0.293	5.387	<0.001	0.687	1.480
	Interaction (X * W)	0.190	3.490	<0.001	0.035	0.127
3	Constant		1.578	0.116	−4.437	40.163
R^2^ = 0.411	RLBs (X)	0.246	4.474	<0.001	0.193	0.498
	BPD symptoms (W)	0.293	5.506	<0.001	0.694	1.468
	Interaction (X * W)	0.212	4.072	<0.001	0.047	0.135

Model 3 was adjusted for gender, age, riding frequency, riding experience, size of motorcycle, status of riding license, status of drinking, history of mental conditions, and perceived peers’ risky riding.

**Table 7:** Hierarchical regression models testing the moderating effect of BPD symptoms on the relationship between RLBs and risky riding behaviors.

Model	Predictors	R^2^	∆R^2^	f^2^	F	*p-*value
1	X, M	0.250	-	-	40.560	<0.001
2	X, M, X * M (interaction)	0.285	0.036	0.049	32.339	<0.001
3	X, M, X * M (with covariates)	0.411	0.126	0.214	13.618	<0.001

Model 3 was adjusted for gender, age, riding frequency, riding experience, size of motorcycle, status of riding license, status of drinking, history of mental conditions, and perceived peers’ risky riding.

In Model 1, both RLBs (*β* = 0.376, *p* < 0.001) and BPD symptoms (*β* = 0.307, *p* < 0.001) were significantly and positively associated with risky riding behaviors, accounting for 25.0% of the variance, supporting H1 and H2.

Model 2 added the interaction term, which was statistically significant (*β* = 0.190, *p* < 0.001) and amounted to a small-to-moderate effect size, in support of H3. The inclusion of this interaction increased the explained variance to 28.5% (ΔR^2^ = 0.036, f^2^ = 0.049).

Model 3 further adjusted for gender, age, riding frequency, riding experience, motorcycle size, driving license status, drinking status, history of mental disorders, and perceived peers’ risky riding. The interaction effect between RLBs and BPD symptoms remained significant (*β* = 0.212, *p* < 0.001). The full model explained 38.1% of the variance in risky riding behaviors (adjusted R^2^ = 0.381).

Multicollinearity diagnostics indicated no violation of assumptions, with all tolerance values above 0.70 and variance inflation factors (VIFs) below 1.50 (see Table 8 for detailed coefficients and collinearity statistics).

**Table 8:** Regression results of predictors of risky riding behaviors.

Predictors	B^1^	SE^2^	*β* ^3^	*p*	95% LL-CI^4^	95% UL-CI^5^	Collinearity statistics	
							Tolerance	VIF
(Constant)	0.762	11.714		0.948	−22.316	23.839		
1. Gender	1.640	1.393	0.063	0.240	−1.104	4.384	0.878	1.138
2. Age	0.464	0.525	0.048	0.378	−0.571	1.498	0.842	1.187
3. Riding frequency	2.104	0.710	0.175	0.003	0.705	3.503	0.726	1.378
4. Riding experience	−0.272	0.618	−0.026	0.661	−1.490	0.947	0.712	1.404
5. Size of motorcycle	0.022	1.090	0.001	0.984	−2.127	2.170	0.958	1.043
6. Status of riding license	1.263	1.546	0.043	0.415	−1.783	4.310	0.930	1.076
7. Status of drinking	0.733	1.330	0.029	0.582	−1.887	3.354	0.914	1.094
8. History of mental disorders	−0.948	2.114	−0.024	0.654	−5.113	3.216	0.859	1.164
9. RLBs^6^	0.346	0.077	0.246	< 0.001	0.193	0.498	0.831	1.204
10. BPD symptoms^7^	1.081	0.196	0.293	< 0.001	0.694	1.468	0.890	1.123
11. RLBs * BPD symptoms (interaction)	0.091	0.022	0.212	< 0.001	0.047	0.135	0.926	1.080
12. Perceived peers’ risky riding	0.530	0.107	0.270	< 0.001	0.319	0.740	0.850	1.177

Adjusted R^2^ = 0.381, F (12, 234) = 13.618, *p* < 0.001.

1 Unstandardized coefficient.

2 Standard error.

3 Standardized coefficient.

4 Lower limit confidence interval.

5 Upper limit confidence interval.

6 Risk-legitimizing beliefs.

7 Borderline personality disorder.

**Figure 2:**
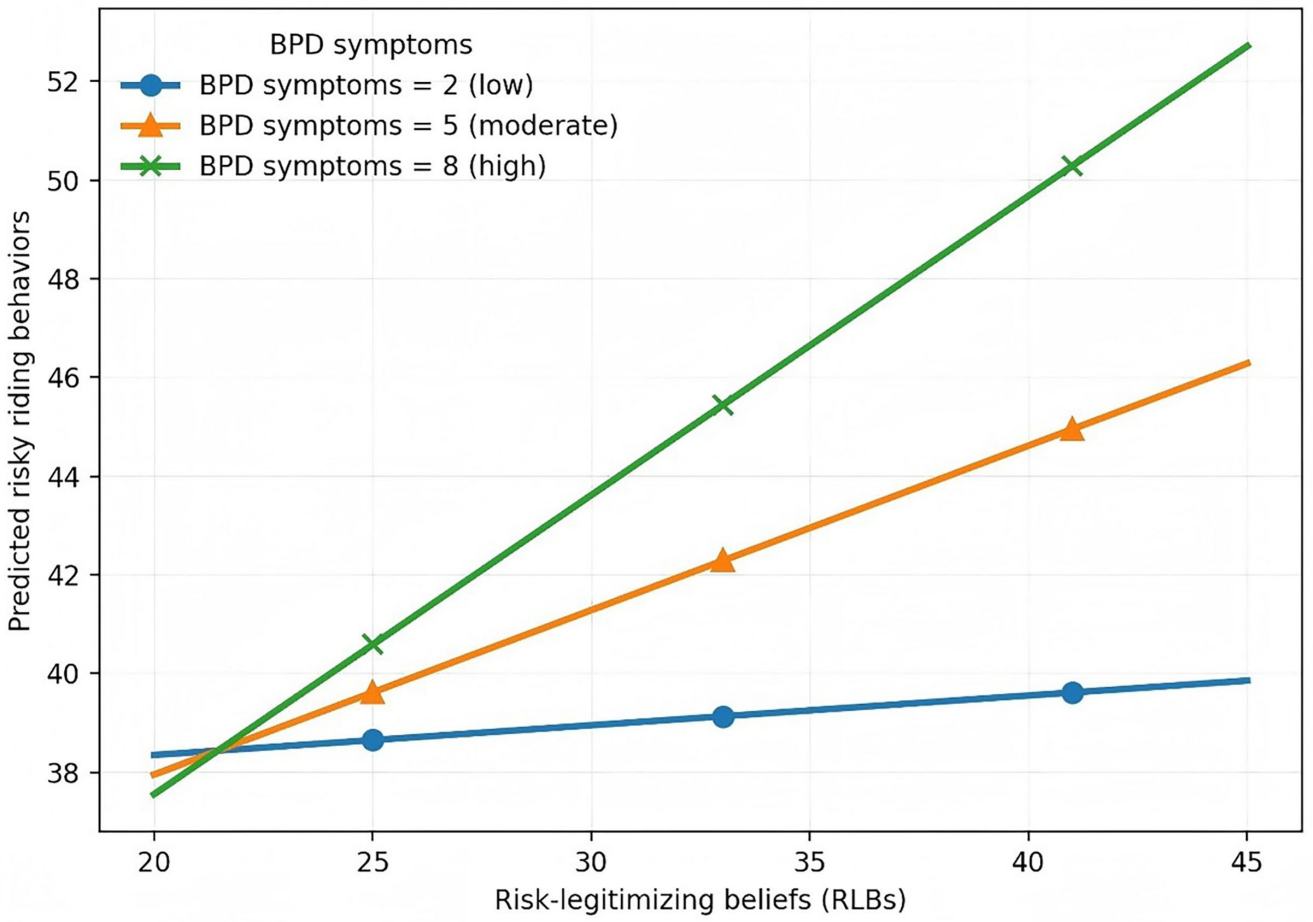
Simple slope plot of the interaction between RLBs and BPD symptoms in predicting risky riding behaviors. Low, moderate, and high BPD symptom levels correspond to observed scale values of 2, 5, and 8 points, respectively.

Simple slopes analysis (Figure 2) indicated that the association between RLBs and risky riding was negligible at low levels of BPD symptoms but became progressively stronger at moderate to high symptom levels. When BPD symptoms were low (2 points), the slope was small and non-significant (*β* = 0.059, SE = 0.108, *p* = 0.585). At a moderate level of BPD symptoms (5 points), the slope was significant and positive (*β* = 0.331, SE = 0.078, *p* < 0.001). At a high level of BPD symptoms (8 points), the positive association between RLBs and risky riding behaviors was strongest (*β* = 0.603, SE = 0.097, *p* < 0.001). This pattern suggests that the practical relevance of the interaction lies not in shifting overall population risk, but in identifying conditions under which the association between RLBs and risky riding is more pronounced.

## 4 Discussion

This study examined how borderline personality disorder (BPD) symptoms moderate the relationship between risk-legitimizing beliefs (RLBs) and risky motorcycle riding among university students in Northern Thailand. Consistent with H1, higher levels of RLBs were associated with higher levels of risky motorcycle riding. In line with H2, higher BPD symptom levels were also independently associated with greater engagement in risky riding behaviors. Regarding the moderation hypothesis (H3), a significant interaction was observed between RLBs and BPD symptoms, indicating that the association between RLBs and risky riding was stronger at higher levels of BPD symptoms. Although this interaction effect was statistically significant, its magnitude was modest, indicating that the association between RLBs and risky riding varies by level of BPD symptoms rather than being uniformly amplified across participants. This effect remained after adjusting for demographic and riding-related covariates, and the full model explained nearly 38% of the variance in risky riding. Together, these findings indicate that cognitive justification and psychological vulnerability are jointly linked to unsafe motorcycle riding among young adults.

Cognitive dissonance theory suggests that individuals may experience discomfort when their behaviors conflict with safety norms and may manage this conflict through various cognitive or behavioral strategies (12). The present study found that higher BPD symptoms were associated with a stronger link between RLBs and risky riding behavior. While these findings align with theoretical accounts suggesting that belief-based rationalizations may be salient for psychologically vulnerable individuals, our study did not directly assess intentions, efforts to change behavior, or specific strategies for dissonance resolution. Therefore, we cannot conclude whether individuals with higher BPD symptoms tend to rationalize behavior rather than alter it. Our results are best interpreted as indicating that the association between belief-based rationalizations and risky riding is more pronounced at higher levels of BPD symptoms, consistent with the possibility that cognitive processes play a greater role in this group. However, this interpretation remains tentative and warrants direct investigation in future research.

Moreover, young riders have been shown to rely heavily on the perceived benefits of risky actions, using a non-compensatory decision process in which salient short-term gains outweigh perceived risks (43). Such benefit-focused reasoning is reinforced by the well-documented time-saving bias, whereby drivers systematically overestimate the time gained by increasing speed and consequently perceive speeding as more worthwhile than it actually is (44, 45). In parallel, the belief that one can “handle the risk” reflects the illusion of control—a cognitive bias in which drivers overestimate their ability to avoid negative outcomes—shown to lower risk perception and increase risky driving behavior (7). Taken together, these forms of cognitive rationalization—amplified by BPD-related impulsivity and emotional instability—may serve as dissonance-reducing strategies that facilitate the maintenance of unsafe riding habits.

Notably, approximately 23.5% of the participants scored above the SI-Bord cut-off (>7), indicating clinically relevant BPD symptom levels even within a nonclinical university sample. This prevalence aligns with prior estimates among student populations (24, 25) and highlights the need to address emotional dysregulation and impulsivity in young adults. Further evidence suggests that these tendencies may be reinforced by underlying neurocognitive mechanisms. Impulsivity—a hallmark of BPD—has been linked to a stronger preference for immediate rewards and reduced sensitivity to potential losses, reflecting difficulties in delaying gratification and in adjusting behavior based on feedback (46–48). Moreover, emotional dysregulation can weaken inhibitory control under stress, as shown by electrophysiological studies indicating that BPD individuals require greater neural effort to maintain control when emotionally aroused (49, 50). These mechanisms may help account for the observed associations between BPD symptoms, cognitive rationalizations, and risky riding under emotionally demanding conditions, such as motorcycle riding.

The observed associations are consistent with previous evidence linking maladaptive beliefs to the persistence of risky or addictive behaviors. Studies in health domains have shown that functional and risk-minimizing beliefs are associated with continued smoking or alcohol use despite awareness of harm (15, 16), supporting the dissonance-reduction account. Extending this framework, research on BPD consistently reports decision-making biases aligned with short-term justifications, which may amplify the impact of RLBs. The current moderation effect suggests that such belief-driven mechanisms may also be implicated in everyday behavioral risks such as motorcycle riding, where emotional self-regulation is essential. Beyond individual cognition, social-cognitive evidence also shows that peer or group norms are among the most reliable predictors of risky driving/riding, particularly among young riders (10, 51). Such external influences may reinforce RLBs by normalizing risky practices within peer groups, thereby being associated with belief–behavior consistency. Taken together, both cognitive and social mechanisms appear to jointly relate to sustained risky riding despite awareness of its dangers.

### 4.1 Theoretical implications

The observed moderation effect suggests that BPD symptoms may be associated with differences in how belief–behavior inconsistency is managed, rather than indicating a uniform mechanism across riders. Specifically, the stronger association between RLBs and risky riding at higher levels of BPD symptoms is consistent with the notion that psychological vulnerability may be linked to a greater reliance on belief-based strategies when dissonance is present. Importantly, this pattern should be interpreted as a conditional association rather than direct evidence of how dissonance is resolved. From a theoretical perspective, these findings help clarify how individual differences in emotional and personality-related vulnerability may shape the strength of the relationship between cognitive rationalizations and unsafe riding, thereby extending cognitive dissonance frameworks into a public mental health context without implying causal pathways.

### 4.2 Practical implications

From a public health perspective, the robust main effects observed in this study indicate that addressing RLBs remains broadly relevant for reducing risky motorcycle riding among university students. Belief-focused strategies—such as brief cognitive-behavioral or psychoeducational exercises directly challenging common rationalizations (e.g., “riding fast does not meaningfully save time”)—may therefore help reduce the cognitive justifications for unsafe behavior at the population level.

At the same time, the modest but significant moderation effect suggests that additional, targeted components may be particularly relevant for riders with elevated BPD symptoms. Incorporating brief mental health screening and embedding short emotion-regulation or mindfulness modules into road-safety education programs may help identify and support riders with elevated BPD symptoms, for whom belief–behavior associations appear stronger.

Finally, peer-based campaigns that reshape perceived norms—highlighting that most students actually value safe riding—may complement individual-level strategies by reducing the social reinforcement of RLBs identified in prior reviews. Taken together, these findings support a feasible, multi-level prevention approach that combines general belief-focused interventions with targeted support for psychologically vulnerable riders, rather than a one-size-fits-all strategy.

### 4.3 Limitations and future research

Several limitations should be acknowledged. Given the cross-sectional design, the observed associations should be interpreted as potentially bidirectional rather than causal. While RLBs may be associated with greater engagement in risky riding, repeated involvement in risky riding may also contribute to the development or reinforcement of such beliefs over time. Similarly, personality-related vulnerabilities such as BPD symptoms may both influence and be influenced by patterns of risky behavior. Longitudinal or experimental studies are therefore needed to disentangle these reciprocal pathways and clarify temporal ordering.

The use of self-report measures and convenience sampling from online university groups may limit representativeness and introduce response bias. As recruitment relied on Facebook student groups, some self-selection bias cannot be ruled out; however, membership verification and inclusion criteria minimized non-student participation. Future studies should complement online recruitment with on-campus sampling to enhance representativeness. Incorporating objective indicators—such as recorded traffic violations or GPS-based riding exposure (e.g., total distance, riding duration, or urban–rural route patterns)—would enhance measurement validity. As the sample consisted predominantly of female university riders in Northern Thailand, the findings should be interpreted with caution in terms of generalizability and external validity. Prior research has documented gender differences in risky riding behaviors and related psychological traits, suggesting that the observed associations may differ in strength or pattern in more gender-balanced or male-dominated samples. Accordingly, the present findings are more directly applicable to female university riders in this context. Future studies should aim to recruit more gender-diverse samples to examine the robustness of these findings across genders. Expanding beyond BPD symptoms to include broader impulsivity- and sensation–seeking–related traits could further clarify when and for whom belief-based mechanisms are most strongly associated with risky riding.

In addition, RLBs were assessed using an adapted scale transferred from health-risk domains such as smoking and drinking, and the present findings should therefore be interpreted as providing initial, exploratory evidence. Although preliminary psychometric analyses supported the use of a unidimensional composite in the current sample, further research is needed to confirm the dimensional structure and measurement invariance of the RLBs scale across populations and contexts.

## 5 Conclusion

Overall, this study showed that risk-legitimizing beliefs (RLBs) and borderline personality disorder (BPD) symptoms were jointly associated with risky motorcycle riding among university students in Thailand. Cognitive dissonance provides a useful conceptual framework for understanding how belief–behavior inconsistencies may be related to the maintenance of risky riding behaviors despite awareness of their dangers. This pattern appears stronger among riders with elevated BPD symptoms. Addressing both belief-based justifications and personality-related vulnerabilities, together with reshaping peer norms, may offer a comprehensive, psychologically informed framework for reducing motorcycle-related risks among university student riders.

## Data Availability

The raw data supporting the conclusions of this article will be made available by the authors, without undue reservation.
